# Soluble Fas ligand released by colon adenocarcinoma cells induces host lymphocyte apoptosis: an active mode of immune evasion in colon cancer

**DOI:** 10.1054/bjoc.2001.2042

**Published:** 2001-09

**Authors:** E Song, J Chen, N Ouyang, F Su, M Wang, U Heemann

**Affiliations:** Department of Medicine, University Hospital Essen, Hufelandstr. 55, Essen, 45122, Germany; Department of Surgery, Sun-Yat-Sen Memorial Hospital, Sun-Yat-Sen University of Medical Science, Guangzhou, 510120, PR China

**Keywords:** soluble Fas ligand, colon neoplasms, apoptosis, immune evasion

## Abstract

Expression of membrane-bound Fas ligand (mFasL) on colon cancer cells serves as a potential mechanism to inhibit host immune function by inducing apoptosis of host lymphocytes. Membrane-bound FasL can be cleaved and released as a soluble mediator (sFasL), which may spread the apoptosis induction effect. Our study examined whether colon adenocarcinoma cells release sFasL, and induce apoptosis of host lymphocytes without direct cell–cell contact. In 12 consecutive patients with colon adenocarcinoma mFasL was identified in the tumours, sFasL was measured in the sera and apoptosis identified in tumour-infiltrating and peripheral blood lymphocytes. To analyse the function of sFasL, colon cancer cells were primarily cultured; sFasL was isolated from supernatants, measured, incubated with Fas-bearing Jurkat cells, and the resulting apoptosis was analysed. Serum levels of sFasL were significantly elevated in all colon cancer patients with mFasL expression in tumour tissues (*n* = 8). In these patients, the number of apoptotic lymphocytes was significantly increased within tumour and peripheral blood. Furthermore, sFasL was present in the corresponding supernatants and induced apoptosis of Jurkat cells in a dose-dependent manner. These findings suggest that mFasL-positive colon cancer cells release sFasL, and thus may induce apoptosis of host lymphocytes as a potential mechanism for immune evasion. © 2001 Cancer Research Campaignhttp://www.bjcancer.com


*British Journal of Cancer* (2001) **85**(7), 1047–1054
© 2001 Cancer Research Campaign
doi: 10.1054/ bjoc.2001.2042, available online at http://www.idealibrary.com on IDEAL

http://www.bjcancer.com

### Soluble Fas ligand released by colon adenocarcinoma cells induces host lymphocyte apoptosis: an active mode of immune evasion in colon cancer

**E Song[1,2], J Chen[2], N Ouyang[1], F Su[2], M Wang[1,2] and U Heemann[1]**

[1]Department of Medicine, University Hospital Essen, Hufelandstr. 55, 45122 Essen, Germany; [2]Department of Surgery, Sun-Yat-Sen Memorial Hospital, Sun-Yat-Sen University of Medical Science, 510120 Guangzhou, PR China

**Summary** Expression of membrane-bound Fas ligand (mFasL) on colon cancer cells serves as a potential mechanism to inhibit host immune function by inducing apoptosis of host lymphocytes. Membrane-bound FasL can be cleaved and released as a soluble mediator (sFasL), which may spread the apoptosis induction effect. Our study examined whether colon adenocarcinoma cells release sFasL, and induce apoptosis of host lymphocytes without direct cell–cell contact. In 12 consecutive patients with colon adenocarcinoma mFasL was identified in the tumours, sFasL was measured in the sera and apoptosis identified in tumour-infiltrating and peripheral blood lymphocytes. To analyse the function of sFasL, colon cancer cells were primarily cultured; sFasL was isolated from supernatants, measured, incubated with Fas-bearing Jurkat cells, and the resulting apoptosis was analysed. Serum levels of sFasL were significantly elevated in all colon cancer patients with mFasL expression in tumour tissues ($n = 8$). In these patients, the number of apoptotic lymphocytes was significantly increased within tumour and peripheral blood. Furthermore, sFasL was present in the corresponding supernatants and induced apoptosis of Jurkat cells in a dose-dependent manner. These findings suggest that mFasL-positive colon cancer cells release sFasL, and thus may induce apoptosis of host lymphocytes as a potential mechanism for immune evasion. © 2001 Cancer Research Campaign http://www.bjcancer.com

**Keywords**: soluble Fas ligand; colon neoplasms; apoptosis; immune evasion

Despite the presence of tumour-specific cytotoxic T cells, the immune system fails to contain colon carcinomas (Luo et al, 1997, 2000). We now know that these malignancies engage a variety of evasive strategies to escape immune clearance, such as aberration of antigen processing and presentation, disruption of T cell–extracellular matrix interactions and immunosuppression by actively modulating and suppressing immune cell functions (Luo et al, 1997; Halapi, 1998). As in other malignant tumours, colon cancer cells actively suppress the immune defence of the host by secreting a number of immunosuppressive factors (Luo et al, 1997, 2000). However, the mechanisms by which such factors overcome anti-tumour immunological responses are still poorly understood.

Recently, it has been demonstrated that a poor prognosis of patients with colorectal carcinomas correlates with a reduced level of tumour-infiltrating and peripheral blood lymphocytes. This reduction may be the result of tumour immunosuppression (Thynne et al, 1979). Fas ligand (FasL), a type II membrane-bound 40-kDa protein which belongs to the tumour necrosis factor (TNF) family, induces apoptotic death of sensitive lymphoid cells expressing its cell surface receptor (FasR; CD95/APO-I) (Nagata and Goldstein, 1995). Activated T and B lymphocytes express FasR, and thus are sensitive to FasR-mediated apoptosis (Krammer et al, 1994; Nagata and Goldstein, 1995). This has been proposed to be responsible for several regulatory functions of the immune system, including tolerance acquisition, down-regulation of immune reactions, and clonal deletion of peripheral lymphocytes (Alderson et al, 1995; Bellgrau et al, 1995; Griffith et al, 1995; Hunt et al, 1997). FasL, which is expressed by indigenous cells of the eye (Griffith et al, 1995) and the testis (Bellgrau et al, 1995), mediates immune privilege by inducing apoptosis in infiltrating pro-inflammatory mononuclear cells. FasL has also been shown to confer immunological privilege in tissue transplantation experiments. Long-term survival was achieved in FasL-expressing allografts (Stuart et al, 1997) and non-immune privileged cells (pancreatic islets) co-transplanted with FasL expressing cells (Korbutt et al, 1997).

FasL expression was also found to be expressed by various malignant tumours, such as oesophageal (Bennett et al, 1998), gastric (Bennett et al, 1999), colonic (O'Connell et al, 1996, 1998), which has been suggested to participate in the potential mechanism of immune evasion in these malignancies (Strand et al, 1996). Membrane-bound FasL (mFasL) may be cleaved by a specific matrix metalloproteinase-like enzyme and be presented in a soluble form (sFasL). Human sFasL is a 26-kDa glycoprotein consisting of an extracellular region for binding to FasR (Tanaka et al, 1996). sFasL was constitutively secreted by prostate cancer cells in vitro (Liu et al, 1998) and detected in the serum of patients with malignant tumours of NK cell origin (Tanaka et al, 1996; Kato et al, 1998). Although it is well established that colon cancer cells express mFasL (O'Connell et al, 1996; Krammer et al, 1998; O'Connell et al, 1998), it remains unknown whether or not they secrete sFasL. Moreover, it remains controversial whether or not sFasL mediates the same effects as mFasL- on FasR-bearing mononuclear cells.

*Received 2 March 2001*
*Revised 11 June 2001*
*Accepted 14 June 2001*

*Correspondence to:* U Heemann

This study focused on the question of whether colon cancer cells bore mFasL, secreted soluble FasL (sFasL) into their local environment and thus induced apoptosis of Fas expressing mononuclear cells without direct cell–cell contact.

### MATERIALS AND METHODS

### Patients

A consecutive series of 12 patients who suffered from colon adenocarcinoma diagnosed by biopsy was examined following a protocol approved by the University Teaching Hospitals ethics committee (5 females and 7 males; ages 47–76, mean age 62.4). All cases underwent radical operation and none of them was found to have metastases in distal organs. Tumours were well ($n$ = 4), moderately ($n$ = 5) and poorly ($n$ = 8) differentiated, including 2 cases of mucinous adenocarcinomas. Pathological staging was performed according to the Dukes system, with 3 cases of Dukes A (mucosa only), 7 cases of Dukes B (into but not through the muscularis propria) and 2 cases of Dukes C (locally positive nodes). Peripheral blood samples were collected one week before and 3 weeks after operation. Sera were isolated and either freshly used or kept in aliquots at –30˚C until used. Mononuclear cells were isolated from the same peripheral blood samples using Ficoll-hypaque gradient centrifugation (Accu-Prep; Accurate Chemical, Westbury, NY, USA). Tissue samples of colon cancers were collected after surgical resection. One part of each sample was used for primary cell culture, the other snap-frozen in 2-methylbutane and stored at –80˚C for further analyses.

### Measurement of sFasL level in sera sFasL levels in sera from 12 healthy volunteers and from all patients 1 week before and 3 weeks after tumour resection were measured with a sFasL enzyme-linked immunosorbent assay (ELISA) kit (Medical and Biological Lab Co, Nagoya, Japan). The kit detects sFasL protein by sandwich ELISA using 4H9 and 4H5 mAbs, anti-FasL monoclonal antibodies against 2 different epitopes. In brief, 96-well plates were incubated overnight with 4H9 mAb (10 µg $ml^{-1}$). Washed with 0.05% Tween PBS (phosphate-buffer saline), the wells were blocked by 2% bovine albumin (Sigma Chemical Co, St Louis, MO, USA) and PBS overnight at 4˚C. 50 µl of the sample, diluted twice with 1% bovine albumin/PBS, was added to the plates and incubated for 1 h at room temperature. Biotinylated anti-Fas monoclonal antibody, 4H5 (5 µg $ml^{-1}$), was then added and incubated for 1 h at room temperature followed by addition of 100 µl of steptavidin-alkaline phosphatase (Life Technologies Gibco BRL, Grand Island, NY, USA). After incubation for 1 h, the plates were developed with 100 µl of 2 mg $ml^{-1}$ p-nitrophenyl phosphate (Life Technologies Gibco BRL, Grand Island, NY, USA). The optical density (OD) was then measured at 450 nm with the use of a microplate reader. The concentration of sFasL was calibrated from a dose–response curve based on reference standards.

### Apoptosis assessment of peripheral mononuclear cells

The number of apoptotic mononuclear cells isolated from peripheral blood was determined by terminal deoxynucleotide transferase-mediated dUTP nick end labelling (TUNEL) assay (Boehringer Mannheim GmbH, Mannheim, Germany) and flow cytometry. Briefly, isolated mononuclear cells were washed in PBS, and permeabilized with 0.1% TritonX-100 in 0.1% sodium citrate solution on ice for 5 minutes. Cells were washed again in phosphate-buffer saline (PBS) and then incubated for 60 min at 37˚C in a labelling TUNEL-reaction mixture containing terminal deoxynucleotidyl transferase (TdT) reaction buffer (10.0 µl), bromodeoxyuridine (Br-dUTP; 8.0 µl), $H_2O$ (32.25 µl), and TdT (0.75 µl). The reaction was then terminated with addition of a rinse buffer. Cells were washed before resuspension in 0.1 ml of PBS. Incorporated Br-dUTP was detected after the addition of fluorescein-labelled anti-bromodeoxyuridine antibody (5.0 µl) and incubation for 30 min at room temperature in the dark. Flow cytometric analysis was performed using a FACScan flow cytometer with LYSIS II software (Nippon Becton Dickinson, Tokyo, Japan).

### Immunohistochemical detection of FasL protein

Standard techniques of immunohistochemistry were used to stain acetone-fixed 4-µm cryostat tissue sections. Endogenous peroxidase activity was quenched by incubation with 3.0% hydrogen peroxide in methanol for 5 min. Sections were washed in PBS and blocked for 1 h in wash buffer containing 5% normal goat serum. A rabbit polyclonal anti-human FasL-specific immunoglobulin G (IgG; Santa Cruz Biotechnology, Santa Cruz, CA, USA) was added as primary antibody at 0.1 µg $ml^{-1}$ in PBS for incubation overnight at 4˚C. After washing with PBS, the sites of primary antibody binding were localized by sequential incubation with biotinylated goat anti-rabbit antibody and then streptavidin conjugated with horseradish peroxidase (LSAB detection kit, Dako Corp, Carpinteria, CA, USA). After further washes in PBS, diaminobenzidine (DAB) was used as a chromogen and sections were lightly counterstained with haematoxylin. In the control section of each case, the peptide immunogen to which the antibody was raised (FasL; N-terminal amino acids 260–279; Santa Cruz Biotechnology) was included at 1 µg $ml^{-1}$ during primary antibody incubation as a direct, internal competitive control for antibody specificity. FasL peptide abolished staining in the tumours.

### In situ apoptosis assessment of tumour-infiltrating lymphocytes

To identify tumour-infiltrating lymphocytes (TILs), CD45 staining was performed on consecutive cryosections with a mouse anti-human CD45 monoclonal IgG (clone 2B11 +PD7/26; Dako Corp, Carpinteria, California, USA; dilution 1/70) according to standard immunohistochemical methods as described above. Apoptosis of TILs was detected in situ in frozen sections of resected tissues by terminal deoxynucleotide transferase-mediated dUTP nick end labelling (TUNEL) assay (Boehringer Mannheim GmbH, Mannheim, Germany) applied to the histological sections. Visualization of the final reaction product was achieved by diaminobenzidine (DAB). TUNEL-positive tumour-infiltrating lymphocytes were counted at high power (× 400) from fields of view chosen according to a systematic random sampling method. Approximately 1000 tumour-infiltrating lymphocytes (TILs) were counted and a TUNEL index (TI) was expressed as the percentage of TUNEL-positive lymphocytes.

### Primary cell culture of colon cancer

Cell suspensions derived from tumour samples were obtained by enzymatic digestion with medium containing 0.1% collagenase

 *© 2001 Cancer Research Campaign*

type 1A, 0.002% DNase type II and 0.05% protease type I (Sigma Chemical Co, St. Louis, MO, USA). Tumour cells were isolated by adherence to plastic culture vessels for 36 h in a 5% $CO_2$ incubator at 37˚C. Supernatants were discarded and tumour cells ($1 \times 10^7$) were cultured in RPMI 1640 supplemented with 10% FCS in a humidified 5% $CO_2$ atmosphere. Assessment of tumour cells was performed by light microscopy, electronic microscopy and immunohistochemistry for keratin.

### Western blot analysis for sFasL in supernatants

Aliquots of supernatants from cultured colon cancer cells were used for Western blot analysis of sFasL. Proteins were isolated by centrifugation (15 000 ***g*** for 20 min twice) and aliquots containing 20 µg of proteins were resolved in 12% SDS-polyacrylamide gel electrophoresis (SDS-PAGE; 12%). The separated proteins were then transferred onto Hybond ECL filter paper (Amersham Life Science, Arlington Heights, IL, USA), which was incubated at 25˚C for 1 h in Blotto A (1 × PBS, 5% milk, 0.05% Tween 20) to block unspecific binding. The filter paper was then incubated at 25˚C for 1 h in Blotto A containing a rabbit polyclonal anti-human FasL specific antibody in 1:200 dilution (Santa Cruz Biotechnology, Santa Cruz, CA, USA). After washing in TBS + 0.05% Tween 20 buffer, filters were incubated for 45 min at 25˚C in TBS + 0.05% Tween 20 buffer containing a 1:10 000 dilution of peroxidase-conjugated secondary antibody (Amersham Life Science, Arlington Heights, IL, USA). To visualize the result, chemiluminescence reaction using the ECL system (Amersham Life Science, Arlington Heights, IL, USA) was performed after washing according to the procedure recommended by the manufacturer.

### Assessment of sFasL-induced apoptosis in Jurkat cell line

Jurkat human T leukaemia cells (American Type Culture Collection, Rockville, MD, USA), which naturally express FasR and are sensitive to Fas-mediated apoptosis, served as target cells to evaluate the pro-apoptotic effect of sFasL containing supernatant. Jurkat cells were cultured in DMEM (Sigma Chemical Co, St Louis, MO, USA) supplemented with 10% fetal calf serum (referred to hereafter as complete medium), in a humidified 10% $CO_2$ atmosphere.

After washing, a total of $5 \times 10^5$ Jurkat cells were incubated for 18 h either with complete media alone or with complete media plus cultured supernatant of colon cancer cells from each case at a ratio of 2:1, 1:1 and 1:2, respectively. In some experiments, target cells were pre-incubated in the presence of the antagonistic anti-FasR blocking antibody clone ZB4 (Biovalley Co., Rockville, MD, USA) at a final concentration of 2 µg $ml^{-1}$ for 1 h. TUNEL assay (Boehringer Mannheim GmbH, Mannheim, Germany) and flow cytometry were performed to quantify apoptosis of target cells as described above.

### Statistical analysis

All experiments for cell cultures were performed at least in triplicate. Results are described as mean ± standard deviation. Statistical analysis was performed by one-way analysis of variance (ANOVA); comparisons between groups were performed by independent sample *t*-test or Bonferroni's multiple-comparison *t*-test. To examine the correlation between serum sFasL level and apoptosis of peripheral blood mononuclear cells, Pearson's correlation coefficient was calculated. A 2-tailed $P$ value less than 0.05 was considered to be statistically significant.

### RESULTS

### mFasL expression in colon carcinomas and apoptosis of tumour-infiltrating lymphocytes

In 8 out of 12 cases (66.7%), membrane-bound FasL (mFasL) was expressed on tumour cells of surgically resected colon cancers as revealed by intense immunohistochemical staining, both, in the cytoplasma and on the surface (Figure 1A). However, intensity and extent of positive staining varied within individual tumours. Neoplastic areas with positive and negative staining for FasL frequently co-existed within the same tumour. Interestingly, homogeneous positive staining was also observed in mucin pools of mucinous carcinomas (Figure 1B), as well as in the intracellular mucin of signet ring cells (Figure 1C). FasL expression also existed on tumour-infiltrating lymphocytes (TILs) in all cases, and the staining was weaker than in positive tumour regions. Additionally, FasL-positive TIL counts were independent of FasL expression on colon cancer cells (data not shown). Fas ligand specificity was confirmed in consecutive control sections with the Fas ligand peptide immunogen as an internal competitive control. Co-incubation with immunogen resulted in direct, competitive displacement of positive staining (not shown) whereas co-incubation with an irrelevant peptide had no effect. No immunostaining was observed in normal mucosal or glandular epithelial cells at the border of the tumours. The extent of mFasL expression did not vary between different cancer stagings or differentiations.

Leukocyte infiltration in tumours was demonstrated in all 12 carcinomas by CD45 immunohistochemical staining. Most of the CD45-positive cells were of lymphoid morphology (not shown). To evaluate apoptosis of tumour-infiltrating lymphocytes, we studied the presence of apoptotic cells in tissue sections by staining of DNA fragments with the TUNEL technique. TUNEL-stained nuclei were primarily found among the infiltrating lymphoctes, both adjacent (Figure 1D) and distal to tumour nests (Figure 1E). Apoptotic cells were detected even among lymphocytes surrounding normal glands close to tumour nests, but without direct physical contact to tumour cells (Figure 1F).

To quantify the extent of apoptosis in lymphocytic infiltrates, the percentage of TUNEL-positive cells was calculated. In mFasL positive colon cancers 2.1 ± 0.9% of infiltrating lymphocytes were TUNEL-positive, as compared to 0.73 ± 0.34% in mFasL-negative cancers ($P = 0.016$) (Figure 2).

### Serum FasL and apoptosis of peripheral blood lymphocytes

Using ELISA, the level of soluble FasL (sFasL) was determined in the serum of colon cancer patients before and after tumour resection. Elevated sFasL levels were only detected in mFasL-positive individuals before tumour resection (Figure 3). One week before tumour resection, sFasL levels of membrane bound FasL-positive individuals ($n = 8$) ranged from 280 to 750 pg $mL^{-1}$ (480 ± 163; 3 patients had sFasL levels exceeding 500 pg $ml^{-1}$). However, 3 weeks after tumour resection sFasL levels were undetectable (< 50 pg $ml^{-1}$) in all patients. sFasL was also undetectable in sera from healthy volunteers

© 2001 Cancer Research Campaign

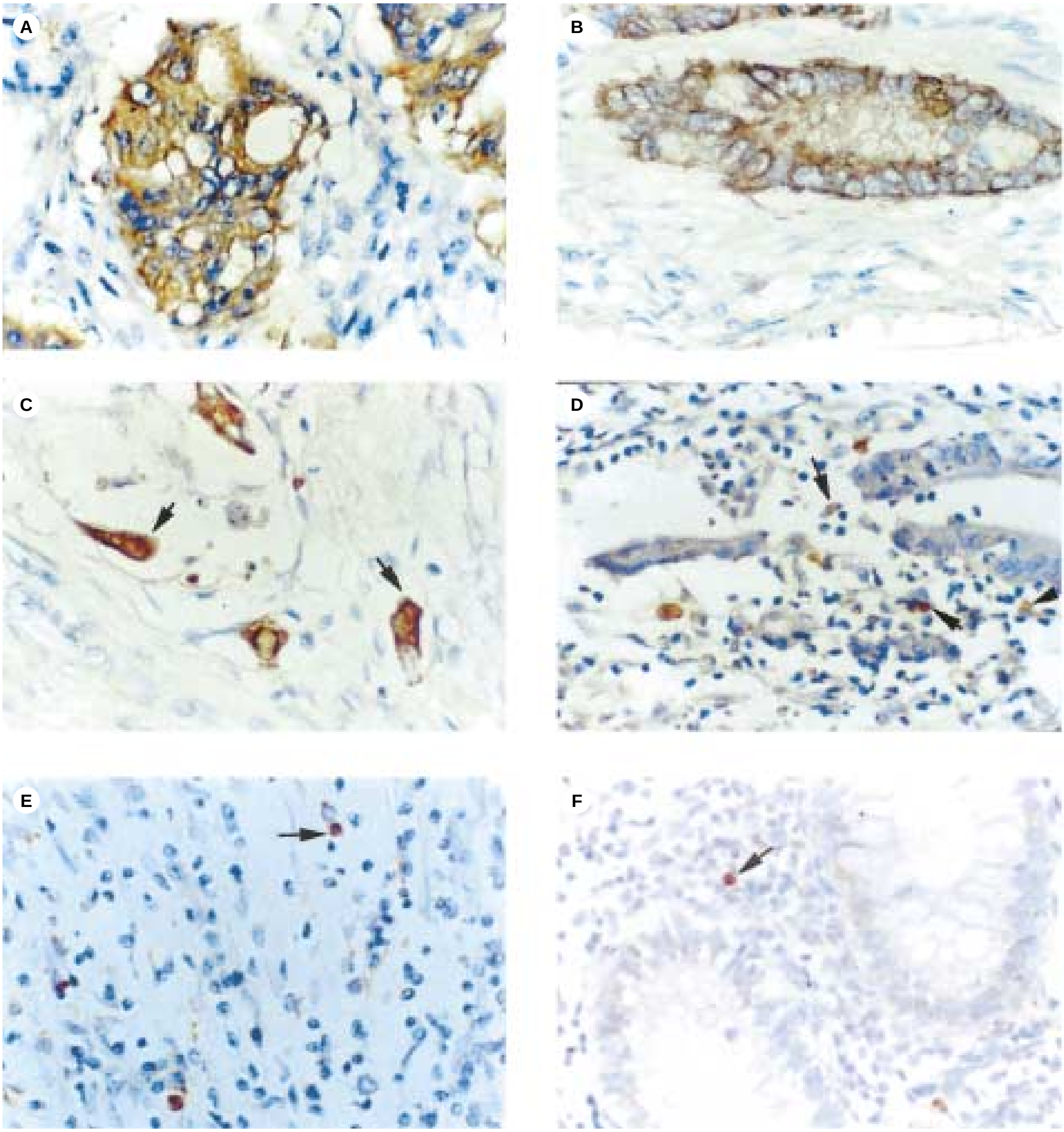

**Figure 1** Fas ligand expression in colon adenocarcinomas (**A**, **B** and **C**) and TUNEL staining of tumour-infiltrating lymphocytes (**D**, **E** and **F**). FasL-positive immunohistochemical staining (brown, × 400) is shown in a representative colon carcinoma (**A**), in the mucin pool of a mucinous carcinoma (**B**), and in the intracellular mucin of signet ring cells (arrowhead) of a mucinous carcinoma (**C**). TUNEL-positive staining (arrowhead) was detected in the infiltrating lymphocytes surrounding tumour nests (**D**, × 400), in the lymphocytes distal to the tumour nest (**E**, × 200), and in those surrounding normal colon glands at the resection rim (**F**, × 400)

and from patients without membrane-bound FasL (mFasL) expression in tumour tissues before or after tumour resection.

There was a significant relationship between sFasL in serum and the number of peripheral blood mononuclear cells (PBMC) undergoing apoptosis (Figure 4) as determined by the TUNEL assay and flow cytometry. In those patients whose sFasL levels were elevated ($n$ = 8), the percentage of apoptotic PBMCs before tumour resection was 0.70 ± 0.20%, and dropped to 0.32 ± 0.11% 3 weeks after tumour resection, at which time sFasL levels were undetectable ($P$ < 0.01). Thus, it was not surprising that the number of apoptotic PBMCs was equally low in cases with undetectable sFasL (0.34 ± 0.17% before and 0.29 ± 0.13% after tumour resection). To further demonstrate the relationship between sFasL levels and the number of apoptotic PBMCs, we conducted a bivariate correlation analysis and calculated the Pearson's correlation coefficient for the 8 cases with detectable serum sFasL. The apoptotic index of PBMCs was positively correlated to serum sFasL levels (r = 0.942, $P$ < 0.01) (Figure 5).

 *© 2001 Cancer Research Campaign*

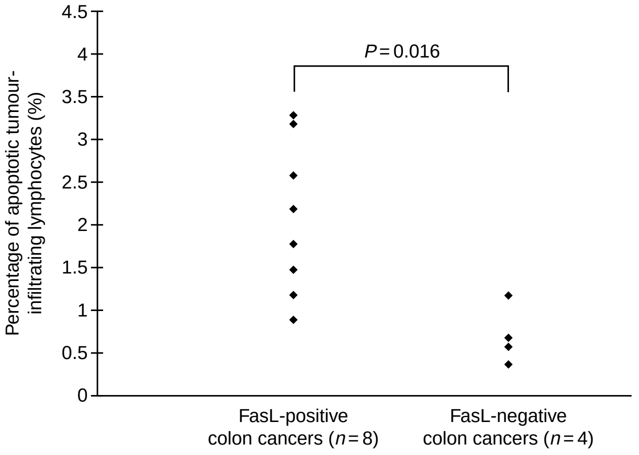

**Figure 2** Percentage of apoptotic tumour-infiltrating lymphocytes (TILs). The percentage of TUNEL-positive tumour-infiltrating lymphocytes (TILs) was calculated by counting TUNEL-positive nuclei among 1000 tumour-infiltrating lymphocytes identified by anti-CD45 mAb immunostaining in consecutive sections

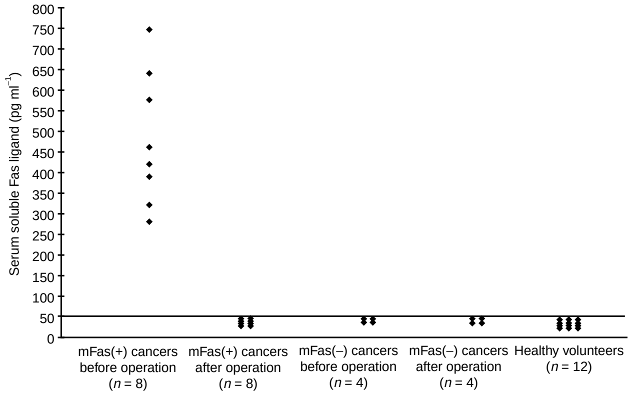

**Figure 3** Serum levels of soluble FasL in patients with colon adenocarcinomas 1 week before and 3 weeks after tumour resection in FasL-positive and in FasL-negative colon cancers, as well as in healthy volunteers

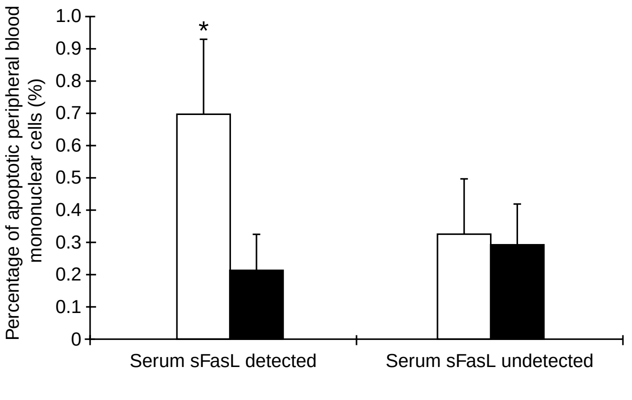

**Figure 4** Percentage of apoptotic peripheral blood mononuclear cells in colon cancer patients before (□) and after operation (■). * denotes significant difference in comparison to the percentage of apoptotic peripheral blood mononuclear cells after operation or to the one of serum FasL undetected group ($P < 0.01$)

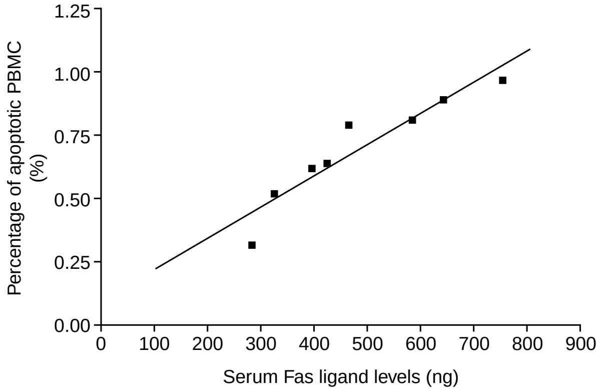

**Figure 5** Bivariate correlation analysis between the percentage of apoptotic peripheral blood mononuclear cells and serum sFasL levels ($r = 0.942$, $P < 0.01$)

### Colon cancer cells release sFasL

To further determine whether colon cancer cells can release sFasL into their local environment, tumour cells were isolated from each resected sample and primarily cultured. Purity of cancer cells, as assessed by light microscopy, electronic microscopy and immunohistochemistry for keratin, was more than 97% in each case (not shown). After 48 h, supernatants were collected and assayed for sFasL. Upon immunoblotting with anti-FasL antibodies, a band migration with an apparent molecular mass of 26 kDa that corresponded to sFasL and represented the cleaved fragment from the 40 kDa mFasL was observed in the 8 cases with positive mFasL expression in tumour tissues and detectable sFasL levels in sera (Figure 6). sFasL was not observed in the supernatants of the other 4 cases without mFasL expression in tumour tissues, as well as in fresh media alone. Besides, in none of the samples could the 40 kDa mFasL be detected in the supernatant.

### sFasL containing supernatants induce apoptosis in Jurkat cells

To test whether sFasL, released by colon cancer cells, can cause Fas-dependent apoptosis of target cells, we incubated Fas-expressing Jurkat cells in media supplemented with supernatants from primarily cultured colon cancer cells at different dilutions. Complete media without supernatant was used as negative control. After 18 h of incubation, the percentage of apoptotic Jurkat cells

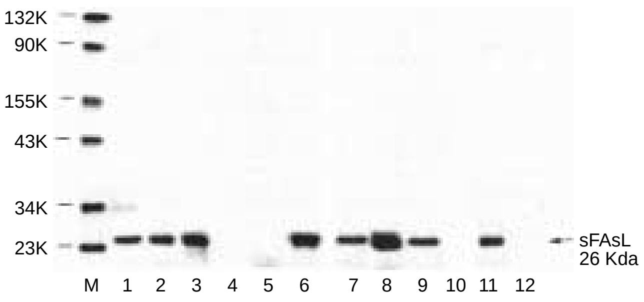

**Figure 6** Western blot analysis of soluble Fas ligand expression in supernatants of primarily cultured colon adenocarcinoma cells developed with anti-FasL antibody. (M: Size marker; 1–12: Patient number). Soluble fas ligand was shown as a polypeptide of 26 kDa in the corresponding 8 cases whose serum sFasL levels were elevated

*© 2001 Cancer Research Campaign*

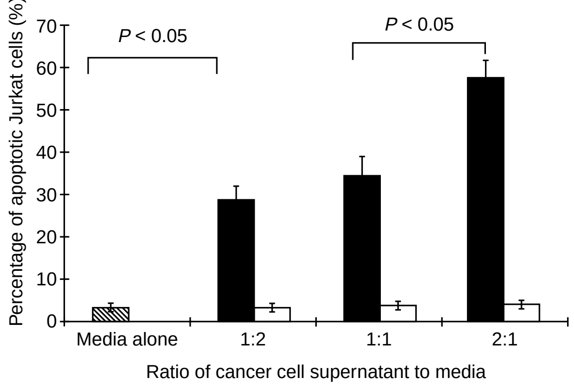

**Figure 7** Percentage of apoptotic Jurkat cells treated with sFasL(+) (■) (*n* = 8) and sFasL(–) (❐) (*n* = 4) supernatants in different ratios of supernatant to media. Jurkat cells treated with media alone (▧) (*n* = 3) served as control exposed to sFasL-containing supernatants was significantly higher than in the ones exposed to complete media or to supernatants without sFasL ($P < 0.05$) (Figure 7). The percentage of cell death increased with the ratio of sFasL containing supernatant to media ($P < 0.05$), being elevated to 28.7 ± 3.5% at a ratio of 1:2 and 57.1 ± 4.3% at a ratio of 2:1. Supernatants without sFasL did not influence the number of apoptotic cells in comparison to complete media.

To determine whether apoptosis of Jurkat cells, exposed to sFasL-containing supernatants, was indeed Fas-mediated, we pre-treated target cells with 2 $\mu g\ ml^{-1}$ of antagonistic anti-FasR-blocking antibody clone ZB4. ZB4 treatment specifically reduced the apoptotic percentage of Jurkat cells exposed to sFasL-containing supernatants at the highest supernatant to media ratio (2:1) (4.8 ± 1.3% vs. 59.7 ± 5.2% in non-treated cells; $P < 0.01$). ZB4 monoclonal antibody alone did not influence the apoptosis of Jurkat cells (data not shown).

### DISCUSSION

Colon cancer cells have been reported to express membrane-bound FasL (mFasL) on their surfaces and thus conduct immune evasion by inducing apoptosis in Fas-expressing lymphocytes (O'Connell et al, 1996, 1998). The requirement of a cell–cell contact was suggested in this type of cell killing (Oyaizu et al, 1997). Recently, the functional soluble form of FasL (sFasL), which consists of the extracellular region of FasL, was identified (Tanaka et al, 1996). The main goal of this study was to determine whether colon cancer cells can release sFasL into host circulation, and whether such soluble extracellular fragment of mFasL may induce apoptotic cell death in host lymphocytes.

We found that high sFasL levels were present in the serum of patients with mFasL-positive colon cancers before tumour resection. High levels of sFasL in serum have been detected in several haematological diseases, including NK-cell leukaemia, NK-cell lymphoma, haemophagocytic lymphohistocytosis, leukaemia of T-cell-type large granular lymphocytes and B-cell non-Hodgkin's lymphoma (Sato et al, 1996; Tanaka et al, 1996; Kato et al, 1998; Murayama et al, 1999; Saito et al, 1999). In these cases, sFasL levels dropped in parallel to the reduced burden of malignant cells following radiotherapy or chemotherapy (Sato et al, 1996; Kato et al, 1998). However, up to now, evidence for the potential of non-lymphoid tumours to release the soluble form of FasL is rare (Liu et al, 1998).

In the present study, the elevated levels of sFasL were reduced to undetectable levels after surgical resection of colon tumours expressing membrane-bound FasL. Furthermore, as we could not detect sFasL in colon cancers negative for membrane bound FasL, sFasL is likely to be released by mFasL-positive colon cancer cells.

More interestingly, in the 2 cases of mucinous adenocarcinomas, which expressed mFasL on the cell surface, we found strong homogenous FasL-specific immunostaining in the extracellular and intracellular mucin secreted or to be secreted by these tumour cells. Since sFasL consists of the extracellular region of mFasL for binding to Fas, it can be detected by immunostaining with anti-FasL immunoglobulin (Liu et al, 1998). Hence, the FasL-specific immunostaining in mucin further supports that colon cancer cells release sFasL. However, it has been demonstrated that sFasL is secreted by activated lymphocytes in inflammation (Toyozaki et al, 1998; Takeda et al, 1999) and graft-versus-host disease (Das et al, 1999). As lymphocytes are present in areas of carcinomas, they might be responsible for the elevated sFasL levels in the sera of colon cancer patients. To testify that colon cancer cells released sFasL, we studied the supernatants of primary cultures for the tumour cells. We could demonstrate that sFasL was present in supernatants of primarily cultured cancer cells from individuals whose serum sFasL levels were markedly elevated, but not in those with undetectable serum sFasL. This confirms that colon cancer cells, rather than TILs, are the major potential source of sFasL.

Lymphocytes in peripheral blood or infiltrating tumour tissues play an important role in anti-tumour immunity. Decrease in tumour-infiltrating and peripheral blood lymphocytes is a characteristic feature of immune evasion in colon cancer (Thynne et al, 1979; Halapi, 1998). The mechanism of lymphocytic apoptosis is not fully understood. Studies on other malignancies have shown a parallel increase in the number of apoptotic tumour-infiltrating lymphocytes (TILs) and mFasL expression on cancer cells, suggesting that tumour cells may kill TILs by a Fas-dependent mechanism (Bennett et al, 1998; Younes et al, 2000). Our data supported this by showing that apoptosis prevailed in TILs of mFasL-positive colon cancers. Furthermore, we found that in addition to a large number of TUNEL-positive lymphocytes surrounding the tumour, lymphocytes that were distal to tumour nests or even adjacent to normal glands near the tumour exhibited a higher percentage of apoptosis than those in mFasL-negative cancers. Since activated T, B and NK cells, neutrophils, and monocytes are sensitive to apoptosis induced by sFasL (Matsumoto et al, 2000; Sporer et al, 2000), the increase in apoptosis of lymphocytes distal to tumour nests, herein, may result from sFasL released by FasL-positive tumour cells.

Accordingly, we also found that the percentage of apoptotic peripheral blood mononuclear cells (PBMCs) was closely correlated with serum sFasL levels. A sharp plunge in the number of apoptotic PBMCs after tumour resection was observed in mFasL-positive colon cancers. This suggests that serum sFasL released by colon cancer cells is biologically active, and induces apoptosis in PBMCs, which may contribute to the immune evasion of colon cancers.

It has been demonstrated by other groups that elevated soluble FasL in serum may cause tissue damages in heart, liver and kidney (Sotozono et al, 1998; Toyozaki et al, 1998; Das et al, 1999; Matute-Bello et al, 1999; Mukai et al, 2000; Sotozono et al, 2000). sFasL present in the bronchoalveolar lavage fluid of patients with ARDS (Matute-Bello et al, 1999) induces apoptosis of cells of the distal pulmonary epithelium during acute lung injury. In our study,

 *© 2001 Cancer Research Campaign*

however, no obvious tissue damage was observed, apart from a slight increase in serum bilirubin levels (higher than 17 μmol $l^{-1}$) in 2 cases with detectable sFasL. The discrepancy between our results and the results of others may be explained by the fact that target cells in damaged organs reported by other groups were activated to express large amount of Fas by concomitant pathogens (Toyozaki et al, 1998).

The role of sFasL in apoptosis induction has been controversial. In-vitro sFasL exerted an anti-apoptotic effect by competing with membrane-bound FasL for its binding to Fas (Oyaizu et al, 1997). Tanaka et al found that release of sFasL down-regulated the expression of mFasL (Tanaka et al, 1998). However, sFasL was also found to exert lethal effects on Fas-bearing cells both in vitro and in vivo (Shiota et al, 1998; Toyozaki et al, 1998; Ghio et al, 1999). In acute self-limited and fulminant hepatitis, serum sFasL levels paralleled the severity of liver damage (Shiota et al, 1998). Besides, serum sFasL in allogeneic blood for transfusion exerts immunosuppression to recipients by inducing apoptosis of Fas-positive lymphocytes (Ghio et al, 1999).

In order to clarify whether apoptosis of peripheral blood and tumour-infiltrating lymphocytes was mediated by sFasL released by colon cancer cells, we evaluated the biological activity of sFasL in the supernatants of primarily cultured colon cancer cells. Massive apoptosis was achieved in Fas-sensitive Jurkat cells treated with sFasL-containing supernatants in a dose-dependent manner, but not in those without sFasL. These effects were abolished when antagonistic anti-FasR blocking antibody was applied. Thus, the apoptosis of Jurkat cells in our study was Fas-mediated.

Our findings suggest that sFasL is biologically active and mediates apoptosis of Fas-bearing lymphocytes. Two possible hypotheses may explain the distinct findings regarding the biological effect of soluble FasL. Gustavo et al proposed that a cofactor may be required for an apoptotic response to sFasL, as higher concentrations of sFasL were required to induce apoptosis in vitro, while a much lower amount was needed in the bronchoalveolar lavage containing cofactors in ARDS subjects (Matute-Bello et al, 1999). This is supported by the observation that colon cancer cells can secrete various kinds of immunosuppressive factors (Luo et al, 1997), which may serve as co-factors for sFasL. Pascal et al suggested that limited mutations in amino acid residues of sFasL result in distinct differences of its biological activity (Schneider et al, 1997). Hence, minor differences in the amino acid sequence of sFasL in cancer cells from other cell types might also explain the different pro-apoptotic effects in different studies.

Overall, our present findings suggest that soluble FasL is released by FasL-positive colon cancer cells, and actively suppresses host immunity by inducing apoptosis of peripheral blood lymphocytes, as well as tumour-infiltrating lymphocytes. Although our previous study has shown that colon cancer cells may be sensitized to FasR-mediated apoptosis upon exposure to cirrhotic Kupffer cells (Song et al, 2001), under normal circumstances, they lack FasR expression, and thus may avoid suicide induced by their own soluble Fas ligand.

### REFERENCES

Alderson MR, Tough TW, Davia-Smith T, Braddy S, Falk B, Schooley KA, Goodwin RG, Smith CA, Ramsdell F and Lynch DH (1995) Fas ligand mediates activation-induced cell death in human T lymphocytes. *J Exp Med* **181**: 71–77

Bellgrau D, Gold D, Selawry H, Moore J, Franzusoff A and Duke RC (1995) A role for CD95 ligand in preventing graft rejection. *Nature* **377**: 630–632

Bennett MW, O'Connell J, O'Sullivan GC, Brady C, Roche D, Collins JK and Shanahan F (1998) The Fas counterattack in vivo: apoptotic depletion of tumor-infiltrating lymphocytes associated with Fas ligand expression by human esophageal carcinoma. *J Immunol* **160**: 5669–5675

Bennett MW, O'Connell J, O'Sullivan GC, Roche D, Brady C, Kelly J, Collins JK and Shanahan F (1999) Expression of fas ligand by human gastric adenocarcinomas: a potential mechanism of immune escape in stomach cancer. *Gut* **44**: 156–162

Das H, Imoto S, Murayama T, Kajimoto T, Isobe T, Nakagawa T, Nishimura R and Koizumi T (1999) Levels of soluble FasL and FasL gene expression during the development of graft-versus-host disease in DLT-treated patients. *Br J Haematol* **104**: 795–800

Ghio M, Contini P, Mazzei C, Brenci S, Barberis G, Filaci G, Indiveri F and Puppo F (1999) Soluble HLA class I, HLA class II, and Fas ligand in blood components: a possible key to explain the immunomodulatory effects of allogeneic blood transfusions. *Blood* **93**: 1770–1777

Griffith TS, Brunner T, Fletcher SM, Green DR and Ferguson TA (1995) Fas ligand-induced apoptosis as a mechanism of immune privilege. *Science* **270**: 1189–1192

Halapi E (1998) Oligoclonal T cells in human cancer. *Med Oncol* **15**: 203–211

Hunt JS, Vassmer D, Ferguson TA and Miller L (1997) Fas ligand is positioned in mouse uterus and placenta to prevent trafficking of activated leukocytes between the mother and the concepts. *J Immunol* **158**: 4122–4128

Kato K, Ohshima K, Ishihara S, Anzai K, Suzumiya J and Kikuchi M (1998) Elevated serum soluble Fas ligand in natural killer cell proliferative disorders. *Br J Haematol* **103**: 1164–1166

Korbutt GS, Elliott JF and Rajotte RV (1997) Cotransplantation of allogeneic islets with allogeneic testicular cell aggregates allows long-term graft survival without systemic immunosuppression. *Diabetes* **46**: 317–322

Krammer P, Dhein J, Walczak H, Behrmann I, Mariani S, Matiba B, Fath M, Daniel PT, Knipping E and Westendorp MO (1994) The role of APO-1 mediated apoptosis in the immune system. *Immunol. Rev* **142**: 175–191

Krammer PH, Galle PR, Möller P and Debatin KM (1998) CD95 (APO-1/Fas)-mediated apoptosis in normal and malignant liver, colon, and hematopoietic cells. *Adv Cancer Res* **75**: 251–271

Liu QY, Rubin MA, Omene C, Lederman S and Stein CA (1998) Fas ligand is constitutively secreted by prostate cancer cells in vitro. *Clin Cancer Res* **4**: 1803–1811

Luo JS, Kammerer R, Schultze H and von Kleist S (1997) Modulations of the effector function and cytokine production of human lymphocytes by secreted factors derived from colorectal-carcinoma cells. *Int J Cancer* **72**: 142–148

Luo JS, Kammerer R, von Kleist S (2000) Comparison of the effects of immunosuppressive factors from newly established colon carcinoma cell cultures on human lymphocyte proliferation and cytokine secretion. *Tumour Biol* **21**: 11–20

Matsumoto J, Kawai S, Terao K, Kirinoki M, Yasutomi Y, Aikawa M and Matsuda H (2000) Malaria infection induces rapid elevation of the soluble Fas ligand level in serum and subsequent T lymphocytopenia: possible factors responsible for the differences in susceptibility of two species of Macaca monkeys to Plasmodium coatneyi infection. *Infect Immunol* **68**: 1183–1188

Matute-Bello G, Liles WC, Steinberg KP, Kiener PA, Mongovin S, Chi EZ, Jones M and Martin TK (1999) Soluble fas ligand induces epithelial cell apoptosis in humans with acute lung injury (ARDS). *J Immunol* **163**: 2217–2225

Mukai M, Bohgaki T, Notoya A, Kohno M, Tateno M and Kobayashi S (2000) Liver dysfunction due to apoptosis in a patient with systemic lupus erythematosus. *Lupus* **9**: 74–77

Murayama T, Koizumi T, Das H, Kobazashi Y, Kajimoto K, Sugimoto T, Imoto S, Nishimura R and Nakagawa T (1999) Soluble fas ligand in natural killer cell lymphoma. *Am J Haematol* **62**: 253–255

Nagata S and Goldstein P (1995) The fas death factor. *Science* **267**: 1449–1456

O'Connell J, O'Sullivan GC, Roche D, Brady C, Kelly J, Collins JK and Shanahan F (1996) The fas counterattack: fas-mediated T cell killing by colon cancer cells expressing fas ligand. *J Exp Med* **184**: 1075–1082

O'Connell J, Bennett MW, O'Sullivan GC, Roche D, Kelly J, Collins JK and Shanahan F (1998) Fas ligand expression in primary colon adenocarcinomas: evidence that the fas counterattack is a prevalent mechanism of immune evasion in human colon cancer. *J Pathol* **186**: 240–246

Oyaizu N, Kayagaki N, Yagita H, Pahwa S and Ikawa Y (1997) Requirement of cell–cell contact in the induction of Jurkat T cell apoptosis: the membrane-anchored but not soluble form of FasL can trigger anti-CD3-induced apoptosis in Jurkat T cells. *Biochem Biophys Res Commun* **238**: 670–675

Saito M, Nakamura N, Nagai M, Shirakawa K, Dato H, Kawahigashi N, Furukawa Y, Usuku K, Nakagawa M, Izumo S and Osame M (1999)

© 2001 Cancer Research Campaign

Increased levels of soluble fas ligand in CSF of rapidly progressive HTLV-1-associated myelopathy/tropical spastic paraparesis patients. *J Neuroimmunol* **98**: 221–226

Sato K, Kimura F, Nakamura Y, Murakami H, Yoshida M, Tanaka M, Nagata S, Kanatami Y, Wakimoto N, Nagata N and Motoyoshi K (1996) An aggressive nasal lymphoma accompanied by high levels of soluble fas ligand. *Br J Haematol* **94**: 379–382

Schneider P, Bodmer JL, Holler N, Mattmann C, Scuderi P, Terskikh A, Peitsch MC and Tschopp J (1997) Characterization of Fas (Apo-1, CD95)-fas ligand interaction. *J Biol Chem* **272**: 18827–18833

Shiota G, Oyama K, Noguchi N, Takana Z, Kitaoka S and Kawasaki H (1998) Clinical significance of serum soluble Fas ligand in patients with acute self-limited and fulminant hepatitis. *Res Commun Mol Pathol Pharmacol* **101**: 3–12

Song E, Chen J, Ouyang N, Wang M, Exton MS and Heemann U (2001) Kupffer cells of cirrhotic rat livers sensitise colon cancer cells to Fas-mediated apoptosis. *Br J Cancer* **84**: 1265–1271

Sotozono C, Sano Y, Suzuki T, Tada R, Ikeda T, Nagata S and Kinoshita S (2000) Soluble Fas ligand expression in the ocular fluids of uveitis patients. *Curr Eye Res* **20**: 54–57

Sporer B, Koedel U, Goebel FD and Pfister HW (2000) Increased levels of soluble Fas receptor and Fas ligand in the cerebrospinal fluid of HIV-infected patients. *AIDS Res Hum Retroviruses* **16**: 221–226

Strand S, Hofmann WJ, Hug H, Muller M, Otto G, Strand D, Mariami SM and Stremmel W (1996) Lymphocyte apoptosis induced by CD95 (Apo-1/CD95) ligand: implications for tumor immune escape. *Nat Med* **274**: 1363–1366

Stuart PM, Griffith TS, Usui N, Pepose J, Yu X and Ferguson TA (1997) CD95 ligand (FasL)-induced apoptosis is necessary for corneal allograft survival. *J Clin Invest* **99**: 396–402

Takeda K, Ohara E, Kaneda T, Hashimoto K and Sasaki M (1999) Increased serum soluble Fas ligand in hyperthyroid Graves's disease. *Rinsho Byori* **47**: 961–965

Tanaka M, Suda T, Haze K, Nakamura N, Sato K, Kimura F, Motoyoshi K, Mizuki M, Tagawa S, Ohga S, Hatake K, Drummond AH and Nagata S (1996) Fas ligand in human serum. *Nat Med* **2**: 317–322

Tanaka M, Itai T, Adachi M and Nagata S (1998) Downregulation of Fas ligand by shedding. *Nat Med* **4**: 31–36

Thynne GS, Moertel CG and Silvers A (1979) Preoperative lymphocyte counts in peripheral blood in patients with colorectal neoplasms: a correlation with tumor type, Kukes' classification, site of primary tumor, and five-year survival rate in 1000 patients. *Dis Colon Rectum* **22**: 221–222

Toyozaki T, Hiroe M, Tanaka M, Nagata S, Ohwada H and Marumo F (1998) Levels of soluble fas ligand in myocarditis. *Am J Cardiol* **82**: 246–248

Younes M, Schwartz MR, Ertan A, Finnie D and Younes A (2000) Fas ligand expression in esophageal carcinomas and their lymph-node metastases. *Cancer* **88**: 524–528

*© 2001 Cancer Research Campaign*
